# Fragmentomics of urinary cell-free DNA in nuclease knockout mouse models

**DOI:** 10.1371/journal.pgen.1010262

**Published:** 2022-07-06

**Authors:** Meihui Chen, Rebecca W. Y. Chan, Peter P. H. Cheung, Meng Ni, Danny K. L. Wong, Ze Zhou, Mary-Jane L. Ma, Liangbo Huang, Xinzhou Xu, Wing-Shan Lee, Guangya Wang, Kathy O. Lui, W. K. Jacky Lam, Jeremy Y. C. Teoh, Chi-Fai Ng, Peiyong Jiang, K. C. Allen Chan, Rossa W. K. Chiu, Y. M. Dennis Lo

**Affiliations:** 1 Centre for Novostics, Hong Kong Science Park, Pak Shek Kok, New Territories, Hong Kong SAR, China; 2 Li Ka Shing Institute of Health Sciences, The Chinese University of Hong Kong, Shatin, New Territories, Hong Kong SAR, China; 3 Department of Chemical Pathology, The Chinese University of Hong Kong, Prince of Wales Hospital, Shatin, New Territories, Hong Kong SAR, China; 4 State Key Laboratory of Translational Oncology, The Chinese University of Hong Kong, Prince of Wales Hospital, Shatin, New Territories, Hong Kong SAR, China; 5 S.H. Ho Urology Centre, Department of Surgery, The Chinese University of Hong Kong, Prince of Wales Hospital, Shatin, New Territories, Hong Kong SAR, China; HudsonAlpha Institute for Biotechnology, UNITED STATES

## Abstract

Urinary cell-free DNA (ucfDNA) is a potential biomarker for bladder cancer detection. However, the biological characteristics of ucfDNA are not well understood. We explored the roles of deoxyribonuclease 1 (DNASE1) and deoxyribonuclease 1-like 3 (DNASE1L3) in the fragmentation of ucfDNA using mouse models. The deletion of *Dnase1* in mice (*Dnase1*^-/-^) caused aberrations in ucfDNA fragmentation, including a 24-fold increase in DNA concentration, and a 3-fold enrichment of long DNA molecules, with a relative decrease of fragments with thymine ends and reduction of jaggedness (i.e., the presence of single-stranded protruding ends). In contrast, such changes were not observed in mice with *Dnase1l3* deletion (*Dnase1l3*^-/-^). These results suggested that DNASE1 was an important nuclease contributing to the ucfDNA fragmentation. Western blot analysis revealed that the concentration of DNASE1 protein was higher in urine than DNASE1L3. The native-polyacrylamide gel electrophoresis zymogram showed that DNASE1 activity in urine was higher than that in plasma. Furthermore, the proportion of ucfDNA fragment ends within DNase I hypersensitive sites (DHSs) was significantly increased in *Dnase1*-deficient mice. In humans, patients with bladder cancer had lower proportions of ucfDNA fragment ends within the DHSs when compared with participants without bladder cancer. The area under the curve (AUC) for differentiating patients with and without bladder cancer was 0.83, suggesting the analysis of ucfDNA fragmentation in the DHSs may have potential for bladder cancer detection. This work revealed the intrinsic links between the nucleases in urine and ucfDNA fragmentomics.

## Author summary

Cell-free DNA (cfDNA) in various bodily fluids, for example blood plasma and urine, is of great importance for noninvasive cancer detection and noninvasive prenatal testing. Many emerging studies on the fragmentation of plasma DNA (i.e., fragmentomics) have received much recent interest. However, the fragmentomics in urinary cfDNA (ucfDNA) remained much less explored. In this study, we explored the biological links between ucfDNA fragmentation and DNA nucleases, using mice for which either the *Dnase1* or *Dnase1l3* gene was genetically inactivated. Interestingly, we found that the deletion of *Dnase1*, but not *Dnase1l3*, caused dramatic alterations of ucfDNA fragmentation patterns, including the elevation of DNA concentration, lengthening of fragment size, disruptions of ucfDNA end motifs (i.e., nucleotide sequences at fragment end) and the jagged ends (i.e., the single-stranded protruding ends). The proportion of fragment ends within DNase I hypersensitive sites (DHSs) was greatly increased in mice with the *Dnase1* deletion. Such ucfDNA fragmentation pattern surrounding DHSs holds potential for classifying the human subjects with and without bladder cancer. The analysis combining various fragmentomic features could further improve the performance for bladder cancer detection, with an AUC of 0.91. This study has shed mechanistic insights into fragmentomics of ucfDNA in urine and has opened up new possibilities for applying ucfDNA fragmentomics in a clinical context.

## Introduction

Cell-free DNA (cfDNA) in plasma has been intensively studied as a class of biomarkers with many diagnostic applications [1], including noninvasive prenatal testing [2–4], cancer assessment [5–8], and organ transplant monitoring [9–11]. cfDNA was nonrandomly fragmented in plasma in such a way that its cleavage patterns were related to nucleosomal packaging [1,12], i.e., the 166-bp major peak with a series of 10-bp periodic small peaks. A number of recent studies focused on mechanistic insights of fragmentomics of cfDNA, including preferred ends, nucleotide motifs at the fragment ends (i.e., end motifs), single-stranded jagged ends, and the relationship between nucleases and cfDNA fragmentation patterns [13,14]. For instance, different nucleases would preferentially generate DNA fragments carrying particular end motifs [15,16]. DNASE1L3 favors cutting cfDNA fragments with the ‘C’ termini, whereas DNASE1 contributes towards the generation of fragments with ‘T’ termini [16]. Furthermore, double-stranded cfDNA molecules were found to commonly possess single-stranded protruding ends [17]. The extent of the jagged ends (i.e., jaggedness) appeared to have association with the sizes of plasma cfDNA molecules and exhibited nucleosomal characteristics.

Compared with cfDNA in plasma, cfDNA in urine exhibited unique fragmentation patterns. For example, ucfDNA molecules were generally more fragmented, with a reduced 166-bp peak but enhanced amplitudes of the 10-bp periodic peaks [18–20]. The jaggedness of ucfDNA was recently reported to be higher than that of plasma cfDNA [21]. However, there is a paucity of mechanistic studies on whether and how the nucleases contribute to ucfDNA fragmentation.

**Fig 1:**
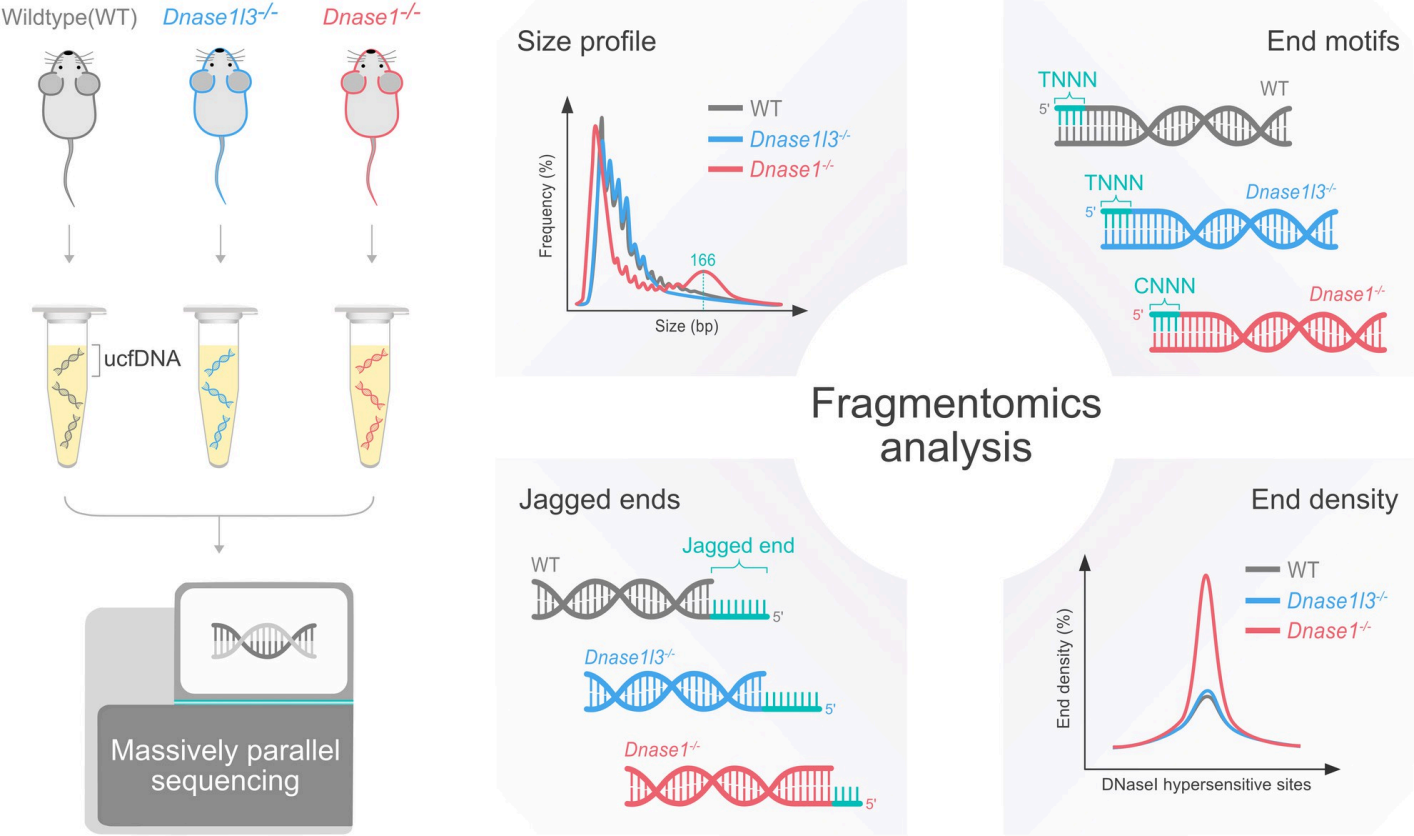
Overview of the study design. Urinary cfDNA was isolated from the urine samples of WT, *Dnase1l3*^-/-^ and *Dnase1*^-/-^ mice and subjected to massively parallel sequencing. Analyses of ucfDNA fragmentation, including the sizes, end motifs, jagged ends and end density across the regions within the DHSs, were performed.

As DNASE1 and DNASE1L3 had been demonstrated to play different roles in plasma DNA fragmentation [15,16], we used mouse models by deleting the *Dnase1* or *Dnase1l3* gene to study their role in ucfDNA fragmentation, including the fragment sizes, end motifs, jagged ends, and end density within DNase I hypersensitive sites (Fig 1). In addition, we explored the potential clinical application of ucfDNA in human subjects with bladder cancer.

## Results

### Concentration of ucfDNA in nuclease-deficient mice

**Fig 2:**
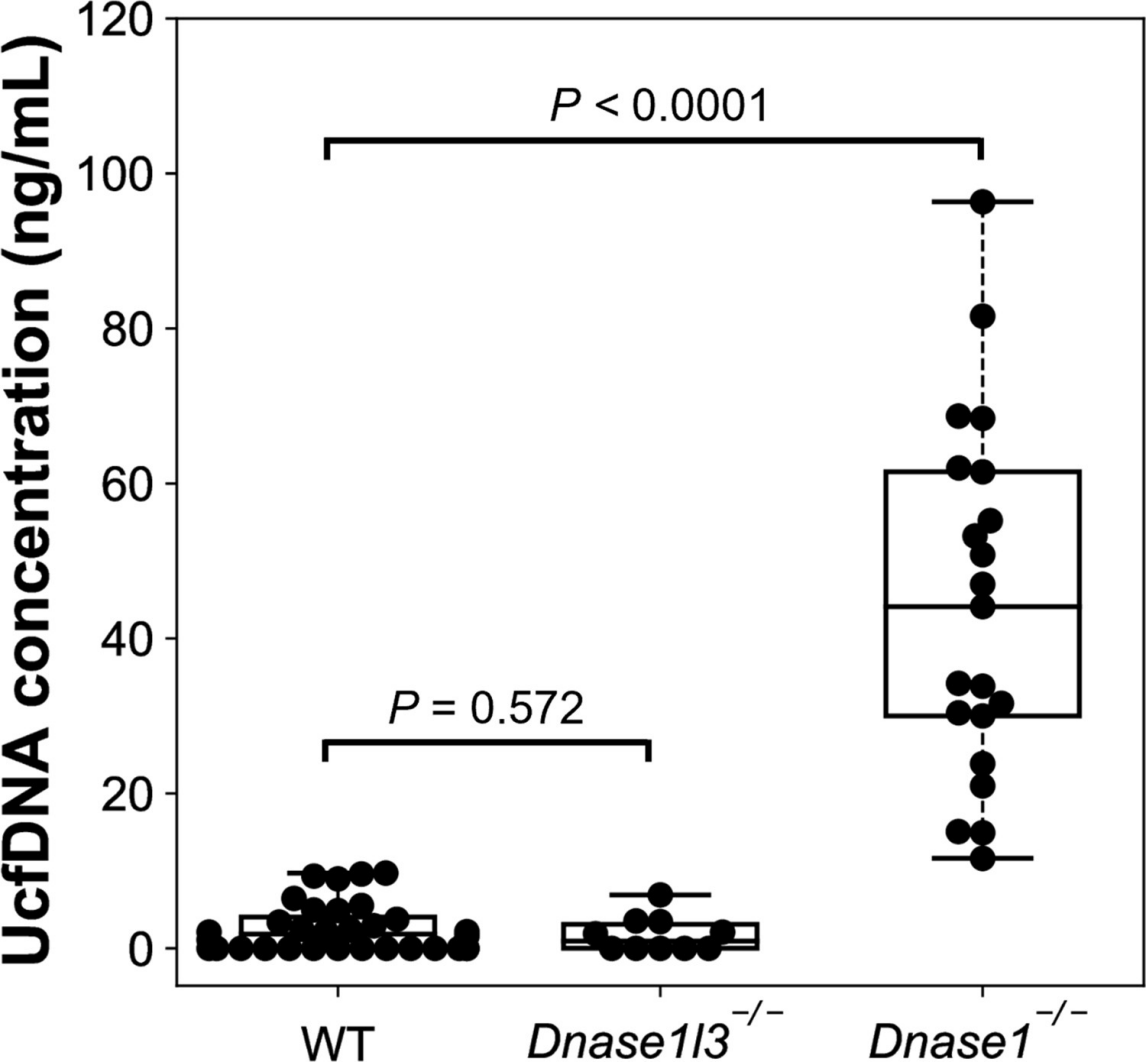
DNA concentrations of ucfDNA in WT, *Dnase1l3*^-/-^ and *Dnase1*^-/-^ mice. DNA concentrations were measured by Qubit and normalized to the corresponding urine volume. Each dot represents a urine sample pooled from three mice.

We analyzed the ucfDNA concentrations of urine samples from wildtype (WT) mice (n = 32), *Dnase1l3*^-/-^ mice (n = 10), and *Dnase1*^-/-^ mice (n = 21) using Qubit dsDNA high-sensitivity assay kit (Thermo Fisher Scientific, Waltham, Massachusetts). The ucfDNA concentration was normalized by the urine sample volume (i.e., ng/mL). The median ucfDNA concentration of the *Dnase1*^-/-^ group (44.1 (range: 11.6–96.3) ng/mL) was 24-fold higher than that of the WT group (1.8 (range: 0–9.7) ng/mL) (*P*-value < 0.0001; Mann-Whitney *U* test). There was no statistically significant difference between *Dnase1l3*^-/-^ (0.9 (range: 0–6.9) ng/mL) and WT groups (*P*-value = 0.572; Mann-Whitney *U* test) (Fig 2). These results suggested that DNASE1 played a role in the degradation of ucfDNA. The conclusion remained the same after the urine samples lacking quantifiable ucfDNA by Qubit dsDNA high-sensitivity assay were removed (S1 Fig).

### Size distribution of ucfDNA among nuclease-deficient mice

**Fig 3:**
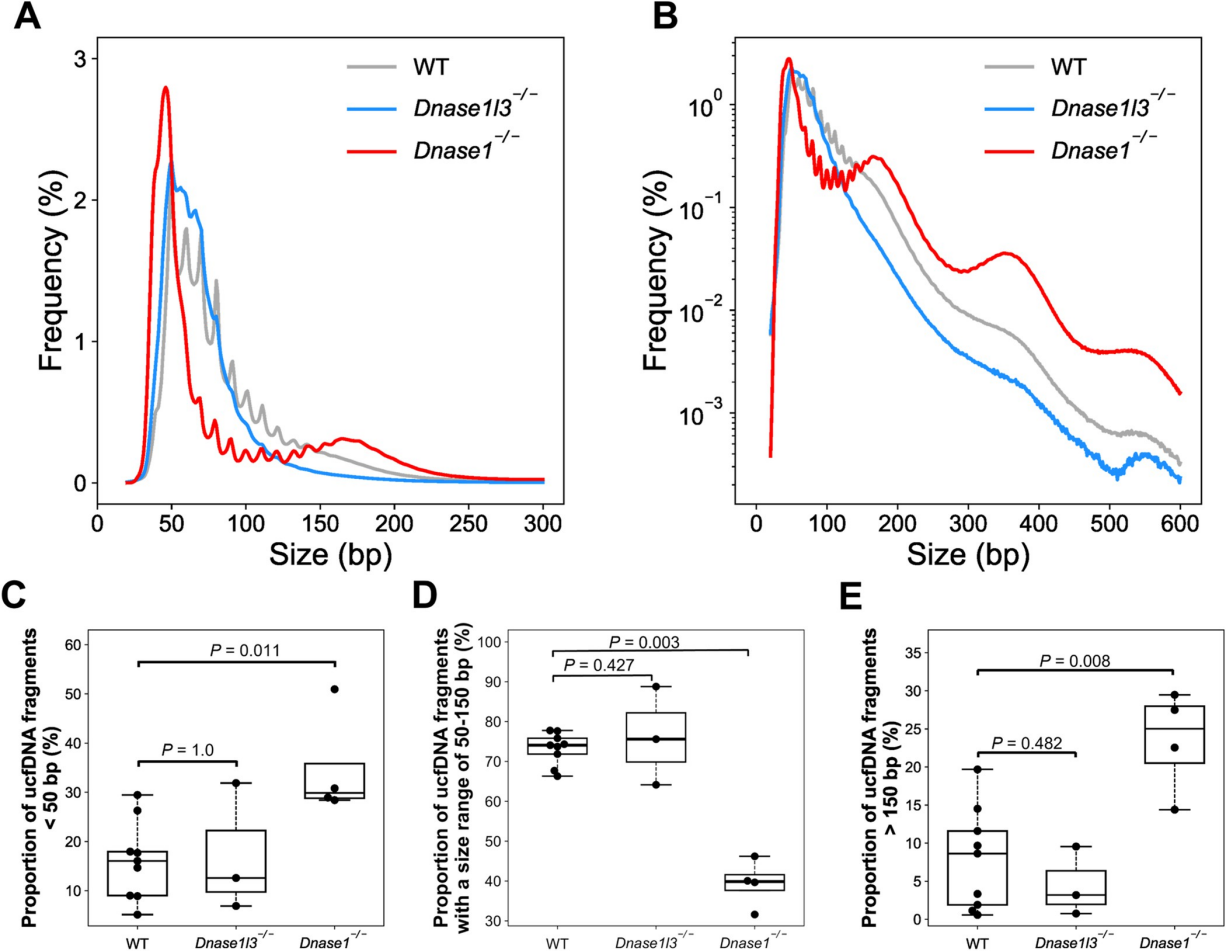
Size distributions of ucfDNA molecules. Size distribution plotted on a linear scale (A), on a logarithmic scale (B) of the y-axis. The grey, blue and red lines represent the median size profile of ucfDNA in WT (n = 9), *Dnase1l3*^-/-^ (n = 3), and *Dnase1*^-/-^ (n = 4) mice, respectively. Boxplots showing the proportion of the fragments < 50 bp (C), ranging from 50 to 150 bp (D), and >150 bp (E).

The overall size profile of ucfDNA from *Dnase1*^-/-^ mice was altered when compared with WT and *Dnase1l3*^*-/-*^ mice. *Dnase1*^*-/-*^ mice had a higher frequency of ucfDNA fragment sizes than WT mice spanning a size range of approximately 20 bp to 50 bp and another size range of around 150 bp or larger (Fig 3A and 3B). The median percentage of fragments with sizes less than 50 bp was 2-fold higher in *Dnase1*^*-/-*^ mice (*Dnase1*^*-/-*^: 29.9%; WT: 16.1%; *P*-value = 0.011; Mann- Whitney *U* test) (Fig 3C), whereas those molecules ranging from 50 bp to 150 bp were significantly reduced in *Dnase1*^-/-^ mice (*Dnase1*^*-/-*^: 39.9%; WT: 74.1%; *P*-value = 0.003; Mann-Whitney *U* test) (Fig 3D). Also, we observed a 3-fold higher in the median percentage of the long DNA fragments (> 150 bp) in *Dnase1*^*-/-*^ mice (*Dnase1*^*-/-*^: 25.0%; WT: 8.6%; *P*-value = 0.008; Mann-Whitney *U* test) (Fig 3E). However, those changes in size were not observed in ucfDNA of *Dnasel13*^-/-^ mice. These data suggested that DNASE1 and DNASE1L3 might play different roles in ucfDNA fragmentation. DNASE1 might be involved in generating the major population of ucfDNA fragments within a size range of 50 to 150 bp.

The size profile of ucfDNA in Fig 3B was plotted on a logarithmic scale for better visualization of the changes in the proportion of the long fragments. The size profile of ucfDNA from *Dnase1*^*-/-*^ mice exhibited increases in the frequencies at mono-, di-, tri-nucleosomal sizes. Such fragments with one or more nucleosomal sizes might be suggestive of internucleosomal cleavage by other nucleases (e.g., DNASE1L3) in the urine of mice with *Dnase1* deletion. The absence of fragments with multiple nucleosomal sizes in the urine of WT mice might be due to highly efficient DNASE1-mediated DNA digestion.

### End motifs of ucfDNA among nuclease-deficient mice

**Fig 4:**
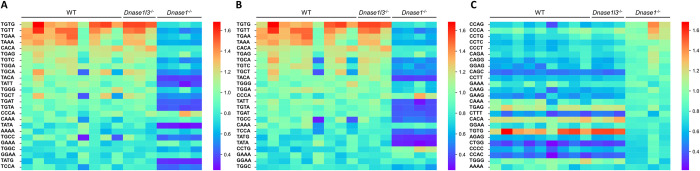
Heatmap showing the end motifs of ucfDNA among different groups. The top 25 4-mer motifs were ranked in descending order according to the frequencies in WT (A), *Dnase1l3*^*-/-*^ (B) and *Dnase1*^-/-^ (C).

The 4-nucleotide oligomer (4-mer) motifs of cfDNA fragment ends could reflect the nuclease activities [22]. We analyzed 256 4-mer end motifs of ucfDNA molecules of different groups. The top 25 4-mer motifs ranked in descending order according to the frequencies in WT (Fig 4A), *Dnase1l3*^*-/-*^ (Fig 4B) and *Dnase1*^-/-^ (Fig 4C) were visualized in the heatmap plots, respectively. Interestingly, the *Dnase1*^*-/-*^ group showed a distinct heatmap pattern as compared to WT and *Dnase1l3*^*-/-*^ groups, while the patterns between WT and *Dnase1l3*^-/-^ groups were similar. We noticed that the abundance of 4-mer motifs beginning with thymine termini (i.e., T ends) decreased in *Dnase1*^-/-^ mice in comparison with WT and *Dnase1l3*^-/-^ mice. Thus, we analyzed fragments with T ends. *Dnase1*^*-/-*^ group showed a significant reduction in the median percentage of the fragments with the ‘T’ ends (24.8%) compared with WT (27.0%) and *Dnase1l3*^*-/-*^ (27.8%) groups (S2 Fig). Hence, DNASE1 might be responsible for generating the T-end fragments in murine urine, which was in concordance with the previous finding in plasma [16].

**Fig 5:**
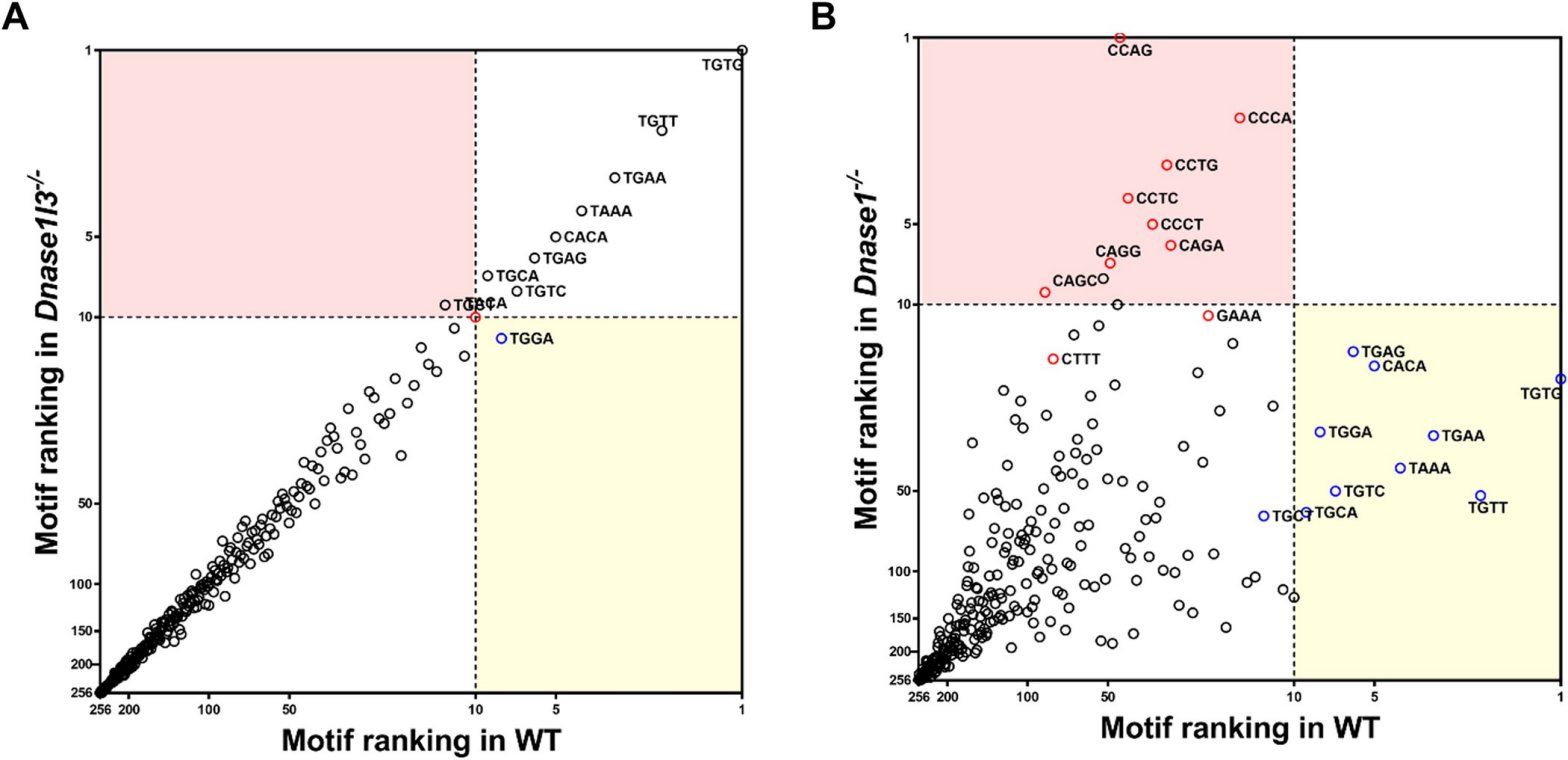
Relationship of motif rankings in ucfDNA between WT and nuclease deficient mice. Correlations of the end motif rankings between WT and *Dnase1l3*^-/-^ groups (A), and WT and *Dnase1*^-/-^ groups (B). Each circle represents a 4-mer motif. Top 10 motifs in WT group were indicated with blue circles, while those derived from *Dnase1l3*^-/-^, and *Dnase1*^-/-^ groups were indicated by red circles.

In Fig 5A and 5B, the rankings of 256 end motifs of the *Dnase1l3*^-/-^ and *Dnase1*^-/-^ groups were plotted against those of the WT group, respectively. The pink area highlighted motifs that were ranked within the top 10 for *Dnase1l3*^-/-^ or *Dnase1*^-/-^ group but ranked 11 or lower for WT group. Conversely, the yellow area highlighted motifs that were ranked within the top 10 for WT group but ranked 11 or lower for *Dnase1l3*^-/-^ or *Dnase1*^-/-^ group. In Fig 5A, a linear relationship between the motif rankings in *Dnase1l3*^-/-^ and WT mice was observed. In contrast, the motif ranking in the *Dnase1*^*-/-*^ was discordant to that in the WT group (Fig 5B). Intriguingly, the rankings of TGTG, TGTT, and TGAA decreased, while that of CCAG, CCTG, and CCCA increased in *Dnase1*^*-/-*^ mice. There were 9 of the 10 motifs in the pink area starting with ‘C’, suggesting that the DNASE1L3 cutting signatures became apparent when DNASE1 was absent.

### Jagged end of ucfDNA among nuclease-deficient mice

**Fig 6:**
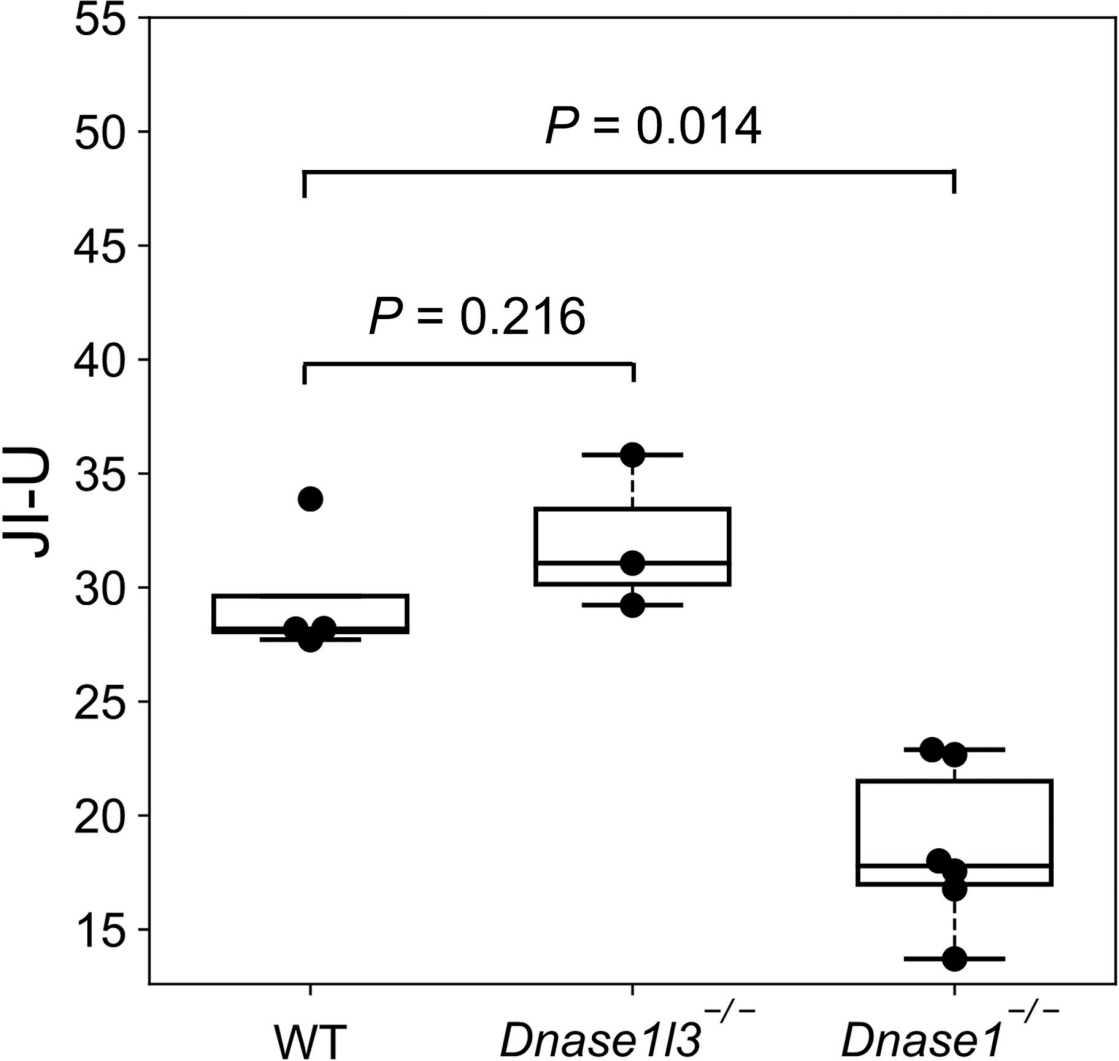
Jagged index-unmethylated (JI-U) values of ucfDNA. Boxplot showing the JI- U values in WT (n = 4), *Dnase1l3*^-/-^ (n = 3), and *Dnase1*^-/-^ (n = 6) groups.

We further studied whether the ucfDNA of mice with nuclease deficiency would affect the generation of jagged ends. The amounts of jaggedness of cfDNA in urine from WT, *Dnase1l3*^*-/-*^ and *Dnase1*^*-/-*^ mice were measured by a metric, which we called Jagged Index-Unmethylated (JI-U). As shown in Fig 6, the median value for JI-U of ucfDNA fragments without size selection in the *Dnase1*^-/-^ group (17.8 (range: 13.7–22.9)) was found to be lower than that of WT (28.2 (range: 27.7–33.9)) (*P*-value = 0.014; Mann-Whitney *U* test) and *Dnase1l3*^-/-^ groups (31.1 (range: 29.2–35.8)) (*P*-value = 0.028; Mann-Whitney *U* test). There was no statistically significant difference between WT and *Dnase1l3*^-/-^ groups (*P*-value = 0.216; Mann-Whitney *U* test). These data demonstrated that the DNASE1 was one of the enzymes responsible for generating jagged ends in ucfDNA.

### UcfDNA fragments at the DNase I hypersensitive sites (DHSs)

**Fig 7:**
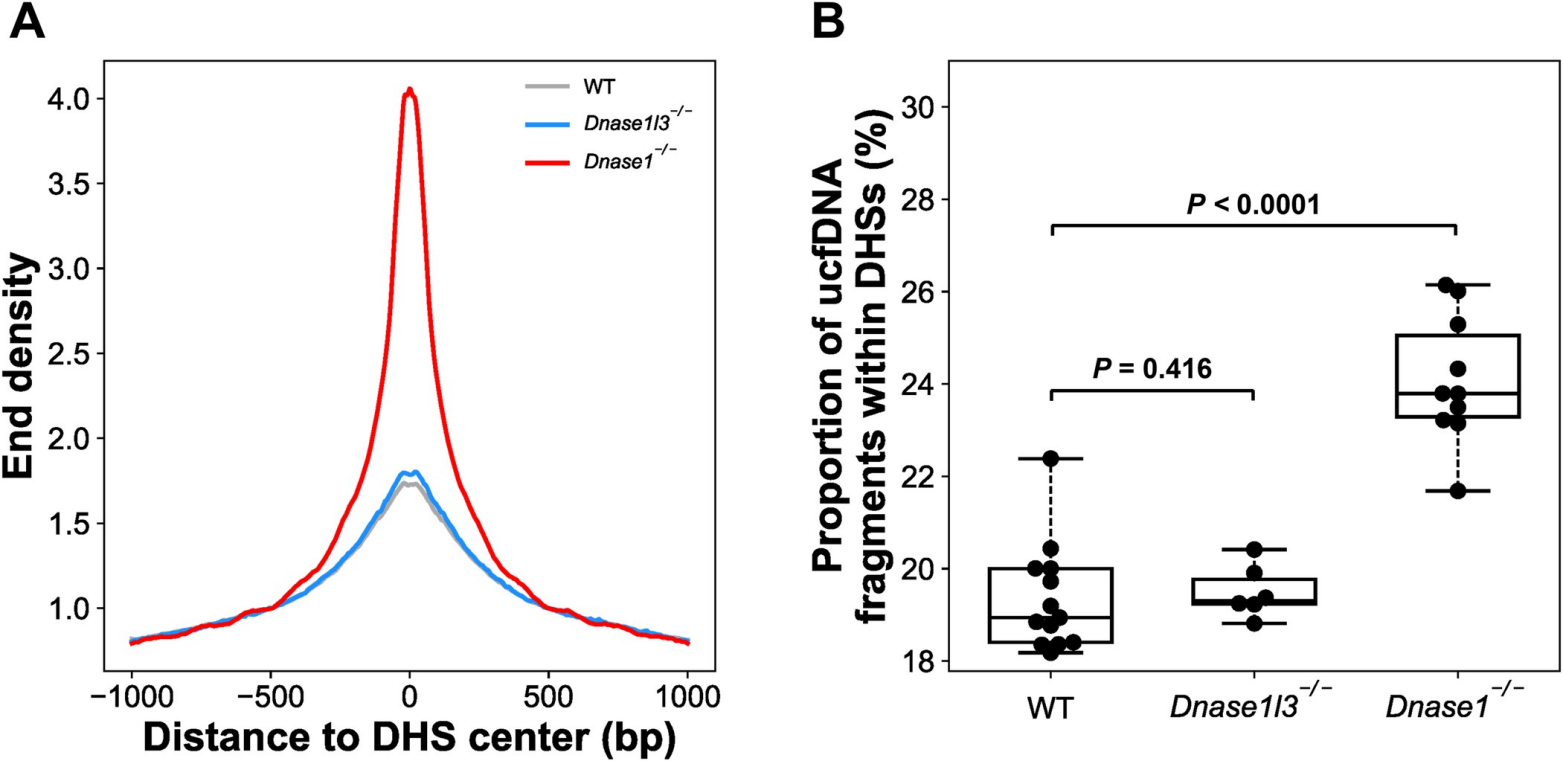
End density of the ucfDNA fragments within the DHSs. (A) End density of ucfDNA fragments across the regions close to the DHSs. The grey, blue and red lines represent the median end density derived from WT (n = 13), *Dnase1l3*^*-/-*^ (n = 6), and *Dnase1*^*-/-*^ groups (n = 10), respectively. (B) Proportion of ucfDNA fragments falling within the DHSs.

As multiple lines of evidence in this study showed that DNASE1 was a key factor in regulating ucfDNA fragmentation, we hypothesized that ucfDNA within the region of the DHSs might be sensitive to DNASE1 activity. In Fig 7A, the normalized end density in DHSs from mouse kidney tissue (0 bp to DHS center) of ucfDNA in *Dnase1*^-/-^ group (4.28) was much higher than the other two groups (WT: 1.71; *Dnase1l3*^-/-^: 1.81). In addition, the *Dnase1*^-/-^ group (23.8% (range: 21.7–26.1%) had a higher proportion of fragments within the DHSs when compared to the WT (18.9% (range: 18.2–22.4%)) and *Dnase1l3*^-/-^ groups (19.3% (range: 18.8–20.4%) (*P*-value < 0.0001; WT vs *Dnase1*^-/-^; Mann-Whitney *U* test) (Fig 7B). However, there was no statistically significant difference between WT and *Dnase1l3*^-/-^ groups (*P*-value = 0.416; WT vs *Dnase1*^-/-^, Mann-Whitney *U* test). Of note, we observed similar differential trends in end density around DHS sites between B cells (S3A Fig) and bladder tissues (S3B Fig) for mice across different groups. It would require a further study to demonstrate whether such end density around DHS sites might bear tissue-specific information with the obtaining of the DHS sites across different tissue types.

### Experimental validation for DNASE1 activity

To explain why DNASE1 is a key driver in the degradation of ucfDNA, which contrasts with blood where DNASE1L3 is the dominant enzyme, we first measured the amounts of DNASE1 and DNASE1L3 protein expression in both healthy human subjects and WT mice using western blot analysis (SDS-PAGE) with a standardized volume (2 μl) of either urine or plasma.

**Fig 8:**
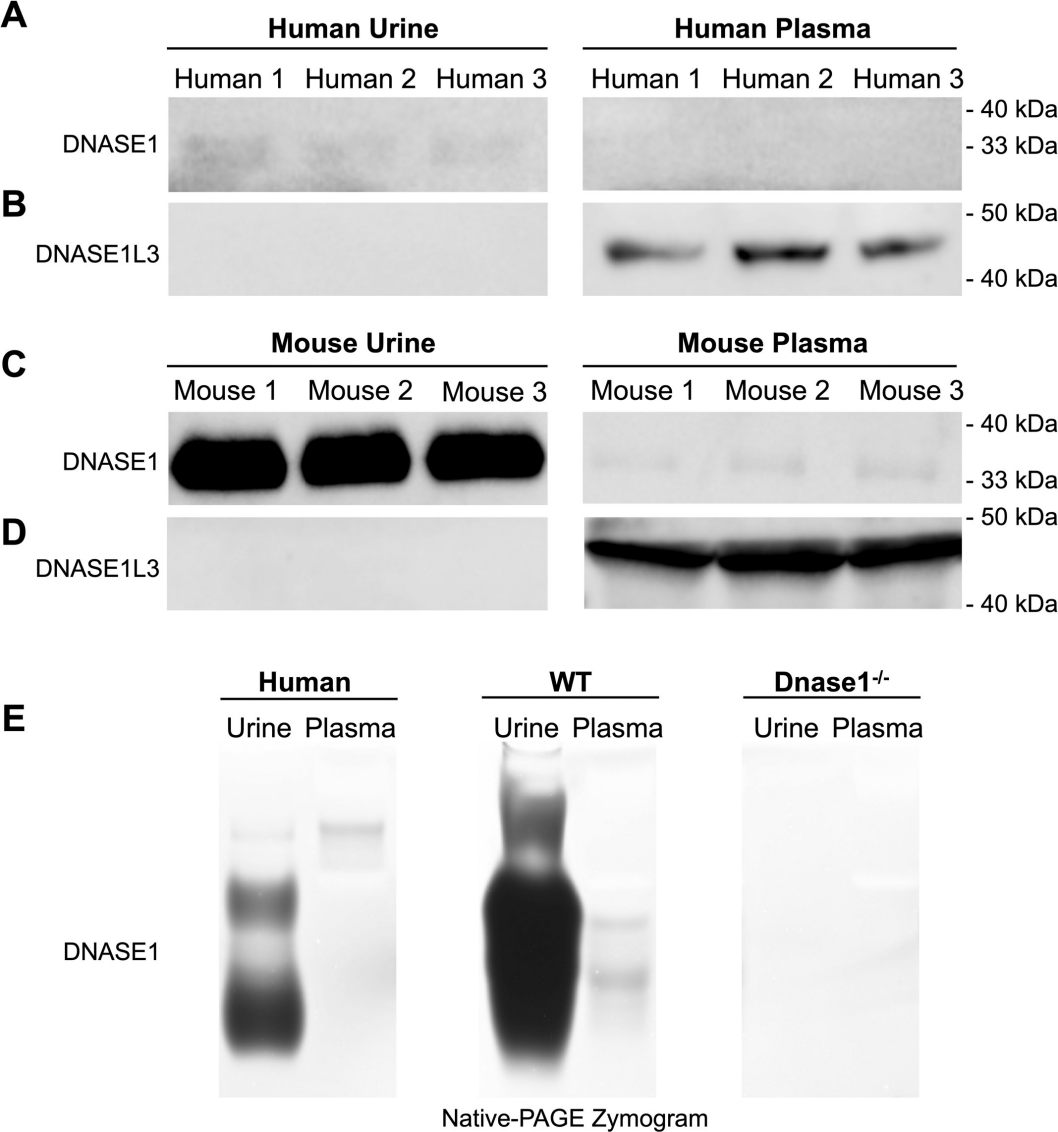
Western blot and zymogram analysis of DNASE1 and DNASE1L3 in urine and plasma samples from healthy human subjects and C57BL/6 mice. Urine (left three) and plasma (right three) samples (2 μl/lane) from three human subjects were subjected to SDS-PAGE and then immunoblotted with anti-DNASE1 antibody (Abcam ab113241) (A) and anti-DNASE1L3 antibody (Abcam ab152118) (B). The same western blot experiment was performed for murine urine (left three) and plasma (right three) samples against anti-DNASE1 antibody (Abcam ab113241) (C) and anti-DNASE1L3 antibody (Abcam ab152118) (D). Urine and plasma samples (2 μl/lane) from human subjects and mice were loaded on the native PAGE gel containing plasmid DNA (pcDNA3.1 Invitrogen) (E). Bands represent the existence of nuclease activity to digest plasmid DNA. Samples from *Dnase1*^-/-^ mice (right) serve as a control. Molecular weight markers (Bio-platform BP106) were used to estimate the sizes of the respective proteins in the immunoblotting. Chemiluminescence detection and gel imaging were performed using ChemiDoc MP Imaging Systems (Bio-Rad Laboratories). The experiments were repeated three times, and one representative experiment with three biological replicates was shown in western blot and zymogram.

Interestingly, in the human samples from three healthy subjects, the amount of human DNASE1 in urine was shown to be greater than that in plasma which was seemingly undetectable (Fig 8A). In sharp contrast, the concentration of DNASE1L3 in plasma (Fig 8B) was shown to be greater than that in urine (Fig 8C). Moreover, we performed the same analysis using murine urine and plasma. The western blot analysis for DNASE1 has shown that the amount of DNASE1 in urine was higher than that in plasma (Fig 8C), consistent with the observation regarding human urine. Conversely, the amount of DNASE1L3 in murine plasma was greater than that in urine (Fig 8D).

Compared with the mouse data (Fig 8C), the differential signal for DNASE1 protein expression in human was relatively low even though a trend for a higher protein expression in urine than in plasma was evident. Hence, we performed the DNASE1 activity assay to compare the DNA-digestion activity of DNASE1 in urine and plasma of the human and mice using a zymogram. Briefly, native polyacrylamide gel electrophoresis (PAGE) was performed on the DNASE1 using a gel containing plasmid DNA (pcDNA3.1; Invitrogen). Instead of using antibodies for protein detection, the trace of digested DNA showing as bands on the gel would be observed, indicating both the activity and the identity of the DNASE1. Samples from *Dnase1*^*-/-*^ mice were also employed for this zymogram experiment to serve as a control. As shown in Fig 8E, the activity of DNASE1 was shown to be higher in the urine than in the plasma for both human and wildtype mice, while the DNASE1 activity was not observed in the urine and plasma of *Dnase1*^*-/-*^ mice. Taken together, we observed a higher DNASE1 protein expression and activity in the urine compared to the plasma in both human and murine samples.

### Potential clinical application of ucfDNA for bladder cancer detection

**Fig 9:**
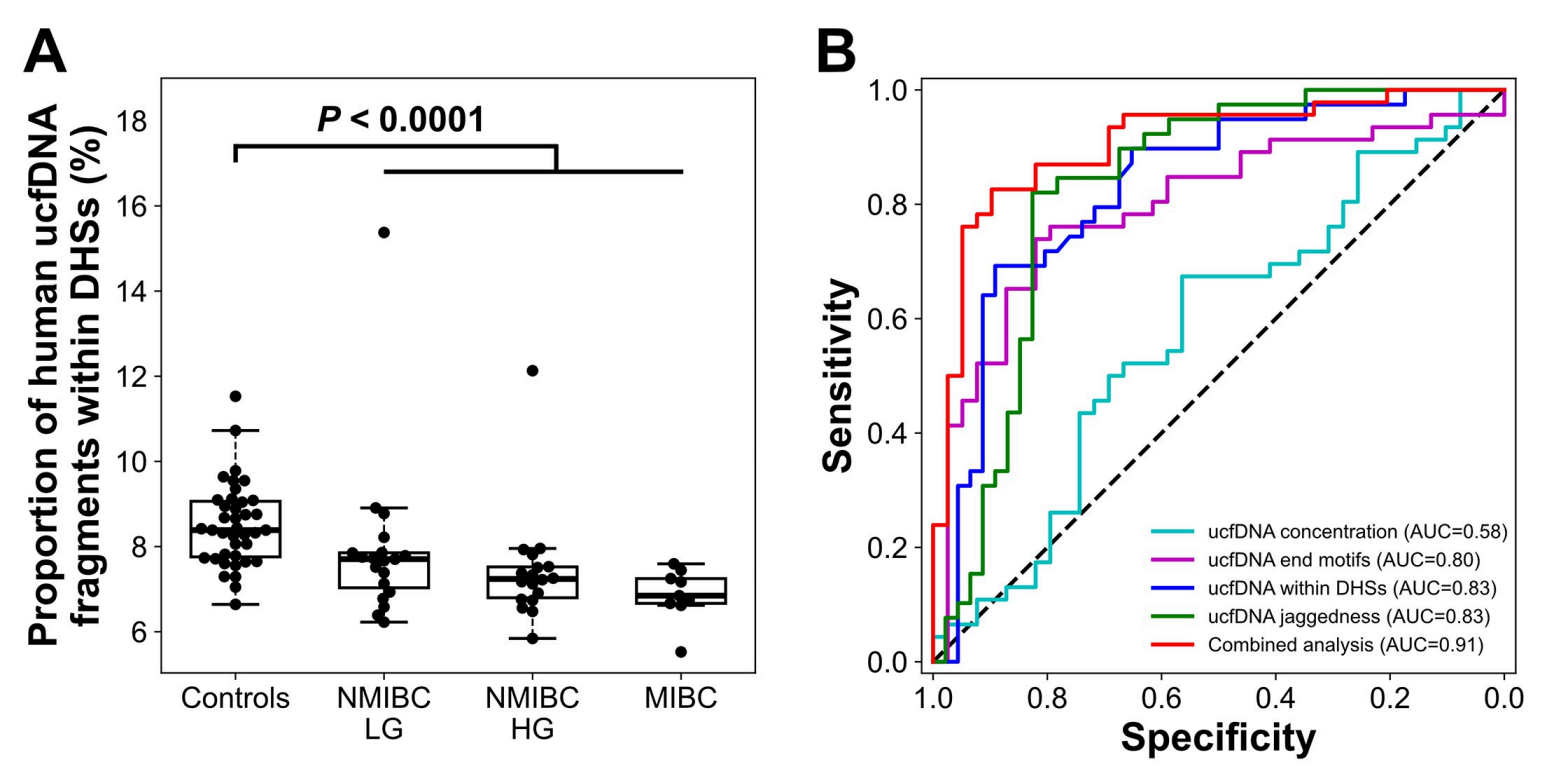
Proportion of human ucfDNA molecules falling within the DHSs. (A) Boxplot showing the proportions of ucfDNA molecules within the DHSs in urine of control subjects with hematuria without bladder cancer (n = 39), and subjects with NMIBC LG (n = 19), NMIBC HG (n = 18), and MIBC (n = 9). (B) ROC plot of differentiation between patients with and without bladder cancer using ucfDNA concentration, jaggedness, end motifs, and the percentage of ucfDNA within DHS.

As we have demonstrated in a previous study that the average contribution of cfDNA from the urinary tract to human urine was substantial (~50%) [19], we reasoned that studying ucfDNA fragmentomics in human participants with urinary tract malignancies would reveal potential applications in cancer diagnostics such as bladder cancer. Additionally, the Cancer Genome Atlas (TCGA) data demonstrated that *DNASE1* mRNA levels were elevated in bladder cancer tumoral tissues as compared to surrounding normal tissues (S4 Fig). In mouse urine from WT mice, a lower fraction of fragments within DHSs was detected (i.e., higher DNASE1 activity), compared to *Dnase1*-deficient animals (i.e., no DNASE1 activity). Thus, it was hypothesized that an increase in DNASE1 activity in bladder cancer tumoral tissues would result in a reduction in ucfDNA molecules produced from DHSs. Indeed, the percentages of ucfDNA fragment ends located within regions 1-kb upstream and 1-kb downstream of DHS (from human kidney tissues) center were significantly decreased in patients with bladder cancer (n = 39) as compared to control subjects with hematuria but without bladder cancer (n = 46) (*P*-value < 0.0001; Mann-Whitney *U* test) (S3D Fig). We also analyzed DHS sites from B cells and observed a decreased trend in end density around DHS sites in patients with bladder cancer, compared with control samples. Such differential trends were shown to be consistent between B cells (S3C Fig) and kidney tissues (S3D Fig). We observed a further decline in the proportion of human ucfDNA fragments within DHSs from kidney tissues in patients with muscle-invasive bladder cancer (MIBC) when compared to subjects with low-grade non-muscle-invasive bladder cancer (NMIBC LG) or with high-grade non-muscle-invasive bladder cancer (NMIBC HG) (Fig 9A). The clinical factors, including age, creatinine, and DNA concentration, showed no significant correlation with respect to the ucfDNA fragments within DHSs in the control or bladder cancer group (S5 Fig).

According to receiver operating characteristic (ROC) curve analysis, the area under ROC curve (AUC) value for differentiating subjects with and without bladder cancer based on the percentage of ucfDNA fragment ends located within the DHSs was 0.83 (Fig 9B). The AUC increased slightly to 0.85 from 0.83 when using ucfDNA molecules within DHSs with a size range of 130–160 bp (S6 Fig). We observed that there was an age difference between controls and bladder cancer patients (*P* value < 0.0001). To evaluate the potential impact of age on the classification analysis, we analyzed ucfDNA within DHSs for those individuals sampled from the original dataset within a comparable age range of 50 to 70 years, forming two age-comparable groups (*P* value: 0.19, Mann-Whitney *U* test) (S7A Fig). We still observed a significantly lower proportion of ucfDNA within DHSs between such age-comparable groups (*P* value < 0.001, Mann-Whitney *U* test) (S7B Fig). The diagnostic performance using ucfDNA within DHSs did not differ between the result based on age-comparable groups (AUC = 0.82) and the original result based on all subjects (AUC = 0.83) (S7C Fig), suggesting that age appeared not to be a confounding factor in this study.

In addition to ucfDNA within DHSs, we further examined other features in ucfDNA including jaggedness [21], end motifs, and ucfDNA concentration (S8 Fig). The metric regarding end motifs was herein referred to as a ratio of two sets of ucfDNA molecules which were tagged by any of the top 25 end motifs identified from WT and *Dnase1*^*-/-*^ mice, respectively. ROC analysis revealed that fragmentomic features including jaggedness, end motifs, and ucfDNA within DHS, with an AUC of at least 0.80 outperformed the metric of ucfDNA concentration [19] (AUC: 0.58) (Fig 9B). The variation of cutoff values across leave-one-out iterations of classification between patients with and without bladder cancers was shown to be small for each metric (S1 Table). We further provided ROC analysis using the ucfDNA concentration normalized by the creatinine concentration (i.e., the ratio of ucfDNA to creatinine concentration) for a subset of samples for which the creatinine measurements were available (38 controls and 21 bladder cancer patients). The diagnostic performance of ucfDNA concentration after the creatinine normalization (AUC = 0.61) did not show any improvement compared to that before the creatinine normalization in the same subset of subjects (AUC = 0.62) (S9 Fig). When these three fragmentomic features were combined using a support vector machine (SVM), one could observe an improved performance in differentiating between cancerous and non-cancerous patients (AUC: 0.91) (Fig 9B). These data indicated that the ucfDNA fragmentomics features may serve as biomarkers for bladder cancer.

## Discussion

In this study, we demonstrated that DNASE1 played a significant role in ucfDNA fragmentation, whereas the impact of DNASE1L3 on ucfDNA fragmentation appeared to be much less significant. The changes in the ucfDNA fragmentation occurring in *Dnase1*-deficient mice included an increase of DNA concentration, an increase of long DNA (>150 bp) with a multinucleosomal pattern (i.e., multiples of 166-bp), a reduction of fragments with the thymine ends as well as the decrease of jaggedness. On the other hand, in plasma, cfDNA from *Dnase1l3*-deficient mice had more significant aberrations in fragment size and end motif frequencies than that of *Dnase1*-deficient mice [15,23].

The different roles of nucleases in urine and plasma might be attributed to multiple factors. First, as shown in the western blot analysis in this study, the concentration of DNASE1 in urine was higher than that in plasma. In addition, the activity of DNASE1 measured by zymography in urine was higher than that in plasma, which was consistent with a previous report [24], possibly partly due to the low concentration of actin in urine that could inhibit the DNASE1 activity [25,26]. Second, the high concentrations of urea and salt in urine might impair the DNA coiling around histones to form nucleosomal structures [27], resulting in reduced protection of DNA from DNASE1 degradation. Further insights into this process might be seen in a recent study that ucfDNA was associated with histone tetramer (i.e., H3_2_H4_2_) instead of intact octamer [20].

The ucfDNA of *Dnase1*^*-/-*^ mice contained two major peaks, the first at 46 bp and the second at 165 bp. We conjecture that ucfDNA is derived primarily from two sources: locally shed ucfDNA from the urinary tract (nontransrenal DNA) and transrenal DNA that passes through the glomerular filtration system [18]. The ucfDNA at the 46-bp peak was reminiscent of the fetal DNA in urine [18], hinting that such a peak might be in part of transrenal origin. DNASE1L3 could digest chromatin DNA into fragments contributing to the peak at 150–160 bp [28]. Hence, we conjecture that DNASE1L3-mediated cleavage of nontransrenal ucfDNA possibly contributes to the presence of ucfDNA fragments around 165 bp in *Dnase1*^*-/-*^ mice. We also observed the multinucleosomal pattern of the ucfDNA in the urine of mice with *Dnase1* deletion, which was reminiscent of the plasma DNA fragmentation pattern caused by DNASE1L3 [15]. Such parallels were further highlighted by the increase of ucfDNA fragments with the cytosine ends in the urine of *Dnase1*-deficient mice. These observations suggested one possibility that the DNA substrates for DNASE1 digestion in urine of WT could be in part originated from the fragments generated by DNASE1L3. Because DNASE1 may predominate in the ucfDNA fragmentation in a highly efficient manner, the DNASE1L3-cutting signature in WT mice urine would be overshadowed. To obtain a more complete mechanistic picture of fragmentation patterns of ucfDNA, a future investigation should include nucleases other than DNASE1 and DNASE1L3.

The cfDNA jaggedness was reported to be higher in urine than in plasma [21]. In the mouse model, we found that the deletion of *Dnase1* led to the reduction of ucfDNA jaggedness, while the deletion of *Dnase1l3* did not cause appreciable difference in ucfDNA jaggedness. These results suggested that DNASE1 might be responsible for the generation of jagged ends of ucfDNA. This finding was consistent with the decreased jaggedness of plasma DNA in the *Dnase1*-deficient mice [17].

DNA organized in cells is well-structured in a way that some regions are relatively more accessible (e.g., euchromatin) and some are less accessible (e.g., heterochromatin) by nucleases. The DHS regions are a set of genomic regions which are supposed to be highly accessible by the DNASE1 enzyme. We reasoned that the cutting frequency within the DHSs would be altered in mice with *Dnase1* deletion. In this study, we observed higher cutting ends of ucfDNA occurring across regions within DHSs in *Dnase1*^-/-^ mice urine. Interestingly, analyzing a bisulfite sequencing dataset in a previous study [19], we found the cutting end frequency of ucfDNA was significantly decreased in patients with bladder cancer compared with controls without bladder cancer. When we made use of ucfDNA end frequency within DHSs for classifying the subjects with and without bladder cancer, an AUC of 0.83 was observed. Thus, such signals of cutting end occurrence within DHSs would be a useful noninvasive biomarker for bladder cancer.

One limitation of this study is that we only sequenced urine samples from mice with detectable ucfDNA concentration using the Qubit assay. All urine samples without quantifiable ucfDNA concentrations were from WT and *Dnase1l3*^*-/-*^ mice. Such a ‘bias’ might be an intrinsic characteristic for urine of WT and *Dnasel13*^*-/-*^ mice because the high activity of DNASE1 is present in WT and *Dnasel13*^*-/-*^ mice. The properties of ucfDNA in such samples with unquantifiable ucfDNA concentrations by the Qubit assay remain unknown and will be studied in a future investigation using enhanced sequencing technology.

In summary, this study unveiled the intrinsic link between nuclease activities and the fragmentation of ucfDNA, providing biological insights into the generation of ucfDNA. This study confirmed that the nuclease-preferred cutting signatures identified from plasma could be generalizable to the urine. Such realization opens up many possibilities to develop biomarkers by utilizing the links between nuclease activities and cfDNA fragmentations in different bodily fluids.

## Materials and methods

### Ethics statement

All animal studies were approved by the Animal Experimentation Ethics Committee of The Chinese University of Hong Kong. The data sets of human subjects were generated following obtaining informed consent from all participants under the approval of the Joint Chinese University of Hong Kong–Hospital Authority New Territories East Cluster Clinical Research Ethics Committee.

### Animals

The *Dnase1l3*^-/-^ and *Dnase1*^-/-^ mouse models were on C57BL/6 (B6) background and were described in a previous study [29]. Wildtype mice on B6 background were used as control and maintained in the same animal facility.

### Sample collection and sample processing

Each urine sample was pooled from the urine of three mice and was collected three times a day for three consecutive days. The urine was collected into a microcentrifuge tube and added with 50 mM EDTA, pH 8.0 (Ambion, Austin, Texas) immediately. The urine was centrifuged at 1,600 g for 10 min at 4°C, followed by another centrifugation at 16,000 g for 10 min at 4°C. All urine samples were kept at -80°C until DNA extraction. We collected a total of 63 urine samples, consisting of 32, 10, and 21 samples from wildtype, *Dnase1l3*^-/-^, and *Dnase1*^-/-^ groups, respectively.

### Data set

The sequencing data of urinary DNA for bladder cancer and control participants (median: 30 million; interquartile range: 19–46 million) was obtained from our previous study [19]. There are 46 patients with bladder cancer (37 non-muscle-invasive bladder cancer and 9 muscle-invasive bladder cancer). All the cancer cases were diagnosed as urothelial cell carcinoma. There are 39 control subjects with hematuria but without bladder cancer. We provided clinical data on the human subjects, including age, gender, degree of bladder cancer invasiveness, and ucfDNA and creatinine concentrations in S2 Table.

### UcfDNA extraction

The ucfDNA was extracted from 2–3 mL of the centrifuged urine using the Wizard Plus Minipreps DNA Purification System (Promega, Madison, Wisconsin). Each 1 mL of urine was mixed with 1.5 mL of 6 M guanidine (Sigma–Aldrich, St. Louis, Missouri). The mixture was incubated with 1 mL of resin for 2 h at room temperature with gentle mixing. The resin-DNA complex was then purified using the Wizard mini-column. The extracted ucfDNA sample was eluted with 70 μL nuclease-free water after centrifugation at 10,000 g for 1 min. Those samples with quantifiable ucfDNA were used for the downstream sequencing library preparation. A total of 44 pooled urine samples had quantifiable ucfDNA by the Qubit dsDNA high sensitivity assay with a Qubit Fluorometer (ThermoFisher Scientific), consisting of 18, 5, and 21 samples from wildtype, *Dnase1l3*^-/-^, and *Dnase1*^-/-^ groups, respectively.

### Library preparation

DNA libraries for the analyses of size distribution and end motif of urine samples were prepared as previously described and subjected to massively parallel sequencing [28]. Briefly, the ucfDNA libraries were constructed using the TruSeq DNA Nano Library Prep Kit (Illumina, San Diego, California) and purified using the MinElute Reaction Cleanup Kit (Qiagen, Hilden, Germany) according to the manufacturer’s instructions. The adaptor-ligated DNA was amplified with fourteen cycles of PCR and then analyzed on an Agilent 4200 TapeStation (Agilent Technologies, Santa Clara, California) using the D1000 ScreenTape System (Agilent Technologies). The libraries were quantified by qPCR using a KAPA Library Quantification Kit (Roche, Basel, Switzerland) on a LightCycler 96 instrument (Roche) before sequencing.

For the jagged end analysis, the libraries were prepared using TruSeq DNA Nano Library Prep Kit (Illumina) with modifications and bisulfite treatment. The ucfDNA was firstly end-repaired with unmodified deoxyribonucleotide triphosphates as previously described [17]. The end-repaired DNA molecules were ligated with methylated sequencing adaptors, followed by two rounds of the bisulfite treatment using the EpiTect Plus Bisulfite Kit (Qiagen). The bisulfite-converted DNA was amplified by 14 cycles with KAPA HiFi HotStart Uracil + ReadyMix (Roche) before the QC validation.

We randomly sequenced a total of 28 pooled mouse urine samples, including 13, 5 and 10 samples with wildtype, *Dnase1l3*^-/-^, and *Dnase1*^-/-^ groups, respectively.

### DNA sequencing and alignment

The ucfDNA libraries were sequenced in a paired-end mode (75 bp x 2) using NextSeq 500 (Illumina). Real-time image analysis and base calling were performed using the NextSeq Control Software v2.1.0 and Real Time Analysis Software v2.4.11 (Illumina) as previously reported [15]. After base calling, adapter sequences and bases with low quality (i.e., quality score < 20) on the 3′ ends of the reads were removed. For the analysis of sequencing data, the sequenced reads were aligned to the non-repeat-masked mouse reference genome (GRCm39/mm39) using the Short Oligonucleotide Alignment Program 2 (SOAP2) [30]. For bisulfite sequencing, the trimmed reads were analyzed using Methy-Pipe software [31]. Only paired-end reads which were uniquely aligned to the same chromosome in a correct orientation, spanning an insert size less than 600 bp were used for further analysis. For reads with the same start and end aligned genomic coordinates, only one would be kept for downstream analysis, whereas the rest were considered as PCR duplicates and were removed. The number of sequenced fragments of DNA library and bisulfite-converted library were summarized in S3 and S4 Table, respectively.

### Jagged index analysis

The jagged index analysis of ucfDNA was performed as described previously [17,21]. Briefly, the JI-U was calculated by the percentage change of methylation density between read 1 and read 2 by the formula:

$$JI-U=\frac{M1-M2}{M1}*100\%$$

where M1 represents the methylation density of 30 bases proximal to the 5’ end, and M2 represents the methylation density contributed by 30 bases proximal to the 3’ end. The higher are the JI-U values, the more jagged ends the ucfDNA would contain.

### Calculation of end density across regions close to DHSs

The DHSs of mouse tissues including kidney (https://www.encodeproject.org/files/ENCFF046NXA/@@download/ENCFF046NXA.bed.gz), B cells (https://www.encodeproject.org/files/ENCFF761OVL/@@download/ENCFF761OVL.bed.gz) and bladder (https://www.encodeproject.org/files/ENCFF073ADQ/@@download/ENCFF073ADQ.bed.gz) were downloaded from the Mouse ENCODE project. The DHSs of human tissues including kidney (https://www.encodeproject.org/files/ENCFF132KNJ/@@download/ENCFF132KNJ.bed.gz) and B cell (https://www.encodeproject.org/files/ENCFF963BED/@@download/ENCFF963BED.bed.gz) were downloaded from Human ENCODE project. UcfDNA fragment ends within the 1-kb upstream and 1-kb downstream of DHSs were analyzed. The end density was defined as the count of fragment ends located within the 1-kb upstream and 1-kb downstream of DHSs divided by the median count across the loci flanking all DHSs.

### Leave-one-out procedure

For the analysis combining different fragmentomics features using SVM, a leave-one-out procedure was used. We excluded one urine DNA sample from 85 human subject samples as a testing sample and used the remaining 84 samples to train a classification model based on SVM. Then, we used this trained SVM model to determine whether or not the testing sample was classified as from a bladder cancer patient. We repeated the aforementioned steps 84 times to validate the model across the remaining samples. To determine the variation of cutoff values for classification between patients with and without bladder cancers for each metric, a similar leave-one-out procedure was adopted as described above.

### Western blot analysis of DNASE1 and DNASE1L3

The DNASE1 and DNASE1L3 levels of the equal volume of urine and plasma samples from healthy human subjects and C57BL/6 mice were compared in western blot analysis. 2 μl of human or murine samples were first diluted to 15 μl with 1 × phosphate-buffered saline (PBS) and mixed with 5 μl 4 × Laemmli sample buffer. The boiled samples were loaded on a 12.5% SDS-PAGE gel and transferred onto a 0.45 μm PVDF membrane (Millipore IPVH00010). The membrane was then immunoblotted with anti-DNASE1 antibody (Abcam ab113241) or anti-DNASE1L3 antibody (Abcam ab152118). Chemiluminescence detection was performed using ChemiDoc MP Imaging Systems (Bio-Rad), and the images were analyzed in Image Lab (Bio-Rad).

### Native PAGE zymogram assay on detecting DNASE1 activity

The DNASE1 activity for urine and plasma samples from human subjects, as well as WT and *Dnase1*^-/-^ C57BL/6 mice, were detected on a 10% native polyacrylamide gel containing plasmid DNA. 10 ml of polyacrylamide gel containing 3.33 ml of 30% Acrylamide/Bis Solution (Bio-Rad, 1610158), 2 ml of 5 × electrophoresis buffer [125 mM Tris, 1250 mM glycine, pH 8.6] and 50 μg pcDNA3.1 plasmid (Thermo Fisher V79020) were polymerized by mixing with 50 μl 10% APS and 8 μl TEMED for 30 mins at room temperature. 2 μl of urine and plasma samples were mixed with an equal volume of 2 × sample loading buffer [25 mM Tris, 250 mM glycine, 50% glycerol, pH 8.6], and loaded on the native PAGE gel without boiling. The gel was run in 1 × electrophoresis buffer at 100 V for 3 hours, and then it was placed into 20 ml of the activation buffer [25 mM Tris, 5 mM CaCl_2_, 5 mM MgCl_2_, 1 × SYBR Gold Nucleic Acid Gel Stain (Thermo Fisher S11494)] in a shaking incubator at 37°C for 2.5 hours. After the activation, the gel was imaged using ChemiDoc MP Imaging Systems (Bio-Rad), and the images were analyzed in Image Lab (Bio-Rad).

### Statistical analysis

Analysis was performed with programs written in Python and R languages. Statistical differences were calculated using Mann-Whitney *U* test. A *P-*value of <0.05 was deemed statistically significant.

## Supporting information

S1 Fig DNA concentrations of ucfDNA across WT, *Dnase1l3*^-/-^ and *Dnase1*^*-/-*^ mice with quantifiable ucfDNA using Qubit assay. (TIF)

S2 Fig Percentages of ucfDNA fragments starting with T. Boxplot showing the percentages of ucfDNA fragments starting with T in WT (n = 9), *Dnase1l3*^-/-^ (n = 3), and *Dnase1*^-/-^ (n = 4) groups.
(TIF)

S3 Fig The end density of ucfDNA across different tissue types. The end density of mouse ucfDNA according to the DHSs from B cells (A) and bladder tissues (B) was determined, respectively. The end density of human ucfDNA according to the DHSs from B cells (C) and kidney tissues (D) was determined, respectively.
(TIF)

S4 Fig Comparison of *DNASE1* mRNA expression levels in bladder tumoral tissues (n = 41) and adjacent non-tumoral tissues (n = 19) using the TCGA dataset. (TIF)

S5 Fig Correlations between the proportions of fragments within DHSs and clinical factors, including ages of human subjects (A), DNA concentration (B), and creatinine concentration in urine (C) in both the control group and bladder cancer group.
(TIFF)

S6 Fig The receiver operating characteristic curve (ROC) for using ucfDNA molecules with a size range of 130 to 160 bp within the DHSs to distinguish control subjects with hematuria from bladder cancer subjects. (TIF)

S7 Fig Analysis of ucfDNA in DHSs between control and age-comparable bladder cancer groups. (A) Boxplot shows that there is no statistically significant difference in age between control subjects and bladder cancer patients. (B) Boxplot of the proportion of ucfDNA within DHSs between two age-comparable groups sampled from the original cohort within an age range of 50 to 70. (D) Comparison between differentiation powers of two age-comparable groups and the originally reported result.
(TIFF)

S8 Fig Boxplots of ucfDNA concentration (A), end motif ratio (B), and jaggedness (C) between controls and patients with bladder cancer.
(TIFF)

S9 Fig ROC analysis for differentiating bladder cancer patients from controls using the ucfDNA concentration before and after normalization by creatinine concentration in those samples with the availability of creatinine measurements (38 controls versus 21 bladder cancer patients). (TIFF)

S1 Table Summary of cutoff values in each iteration of leave-one-out analysis for classifying patients with and without bladder cancers using different metrics. The metrics include the proportion of ucfDNA within DHSs, ucfDNA jaggedness, and ucfDNA end motifs.
(DOCX)

S2 Table The clinical data of controls and bladder cancer patients. (DOCX)

S3 Table The number of sequenced reads of each sample using massively parallel sequencing. (DOCX)

S4 Table The number of sequenced reads of each sample using Bisulfite sequencing. (DOCX)
