# Supplementary figures and images for: Fragmentomics of urinary cell-free DNA in nuclease knockout mouse models
Source: PLoS Genet. 2022 Jul 6;18(7):e1010262. doi: 10.1371/journal.pgen.1010262 (PMC9258866; doi:10.1371/journal.pgen.1010262)

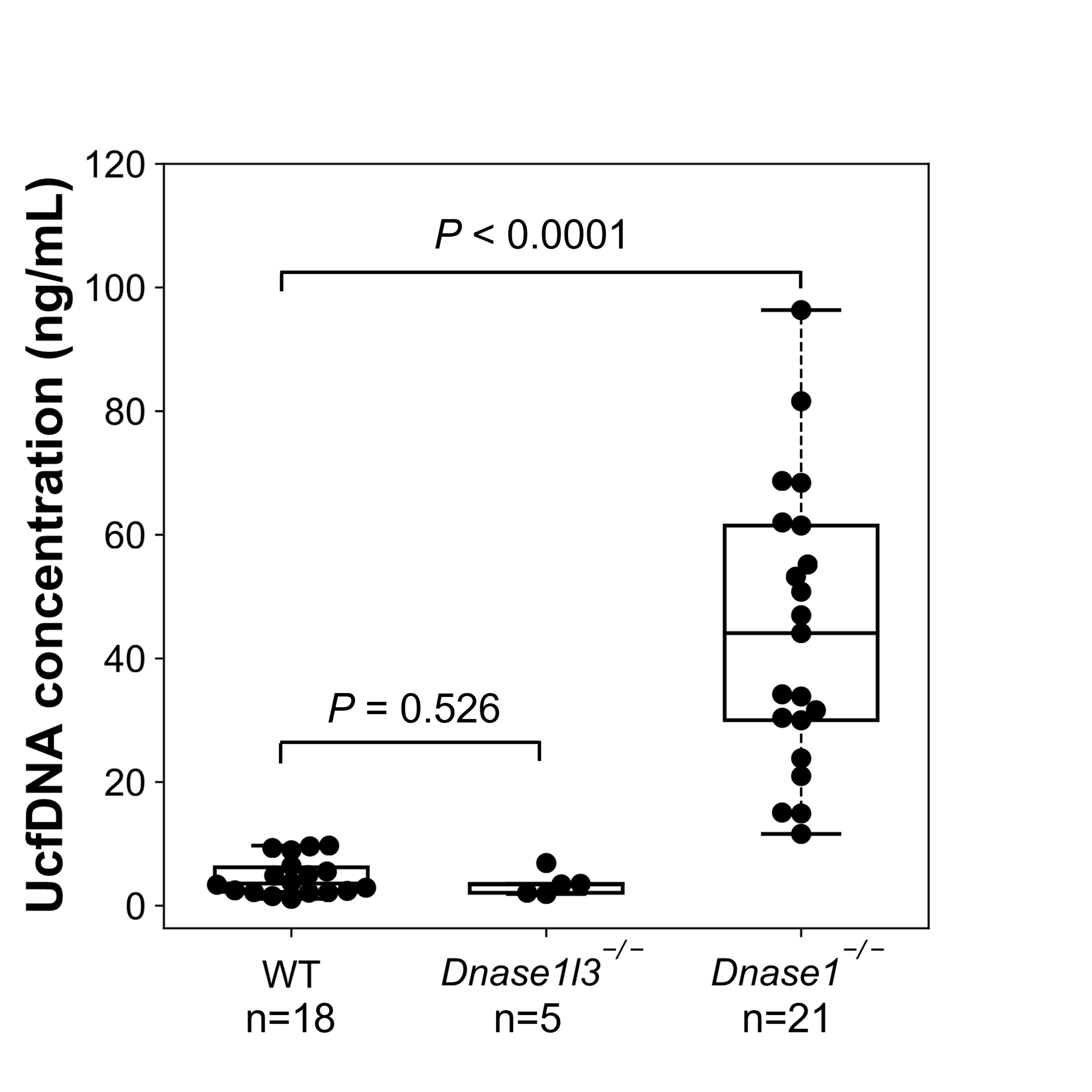

Supplement: S1 Fig — (TIF) [file pgen.1010262.s001.tif]

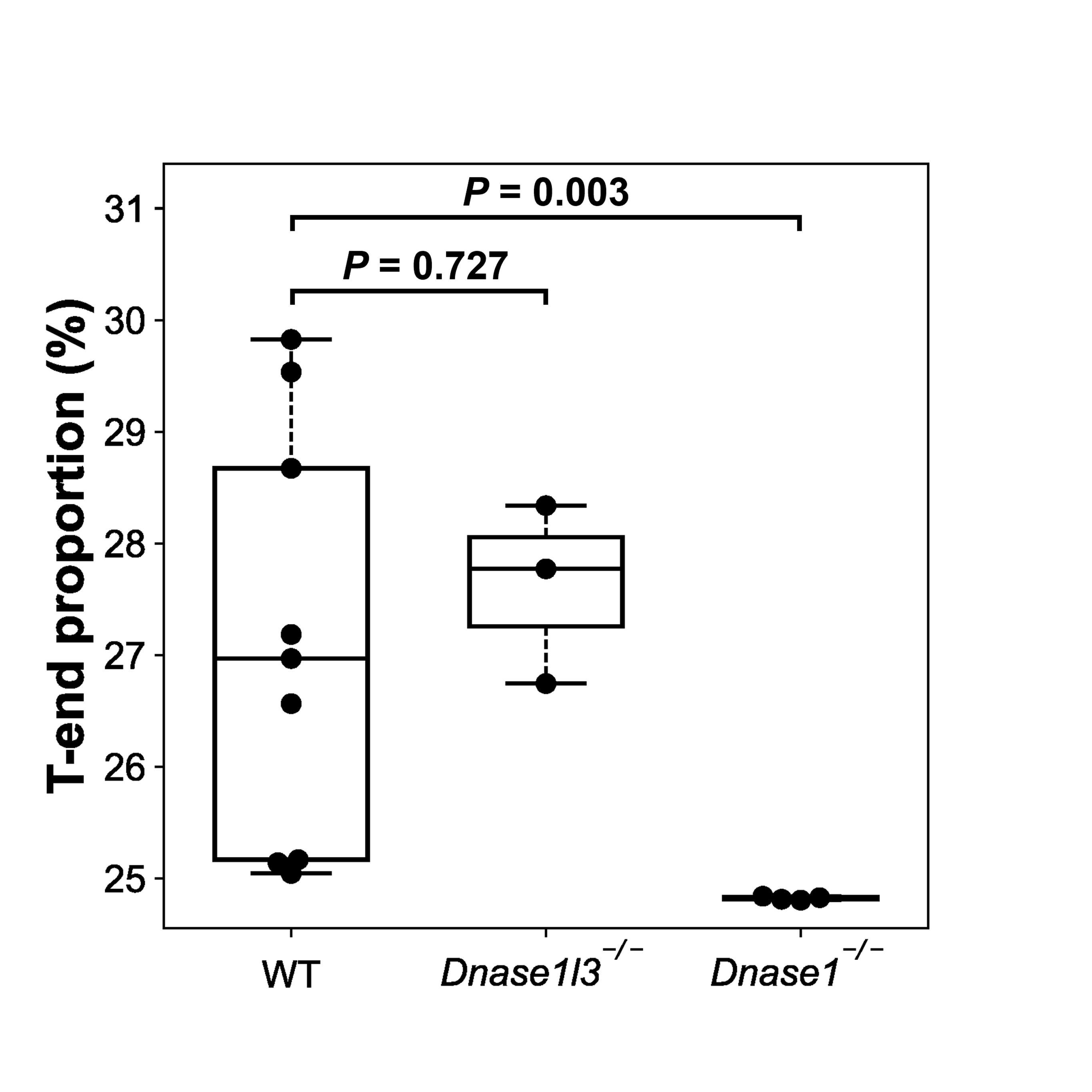

Supplement: S2 Fig — Boxplot showing the percentages of ucfDNA fragments starting with T in WT (n = 9), Dnase1l3-/- (n = 3), and Dnase1-/- (n = 4) groups. (TIF) [file pgen.1010262.s002.tif]

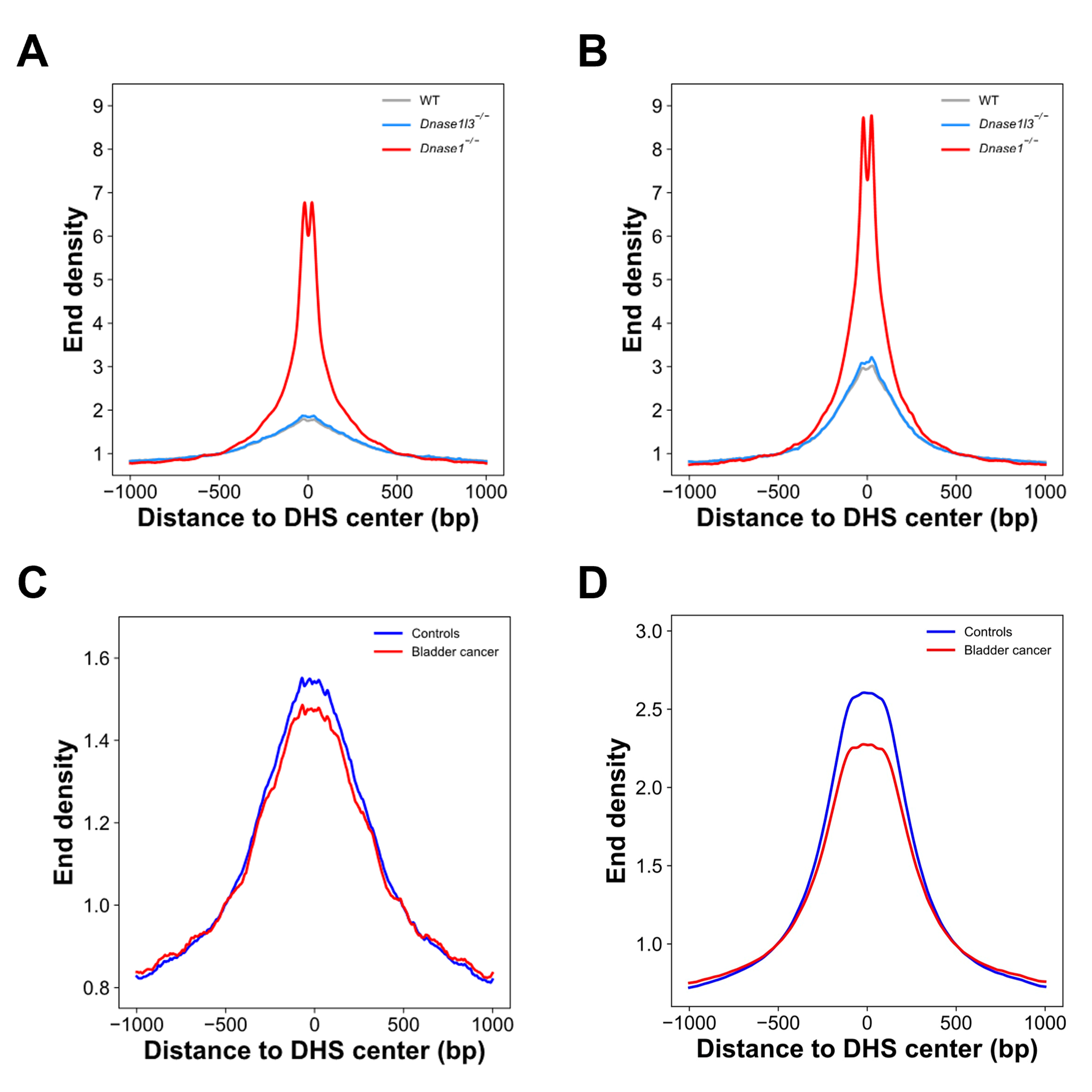

Supplement: S3 Fig — The end density of mouse ucfDNA according to the DHSs from B cells (A) and bladder tissues (B) was determined, respectively. The end density of human ucfDNA according to the DHSs from B cells (C) and kidney tissues (D) was determined, respectively. (TIF) [file pgen.1010262.s003.tif]

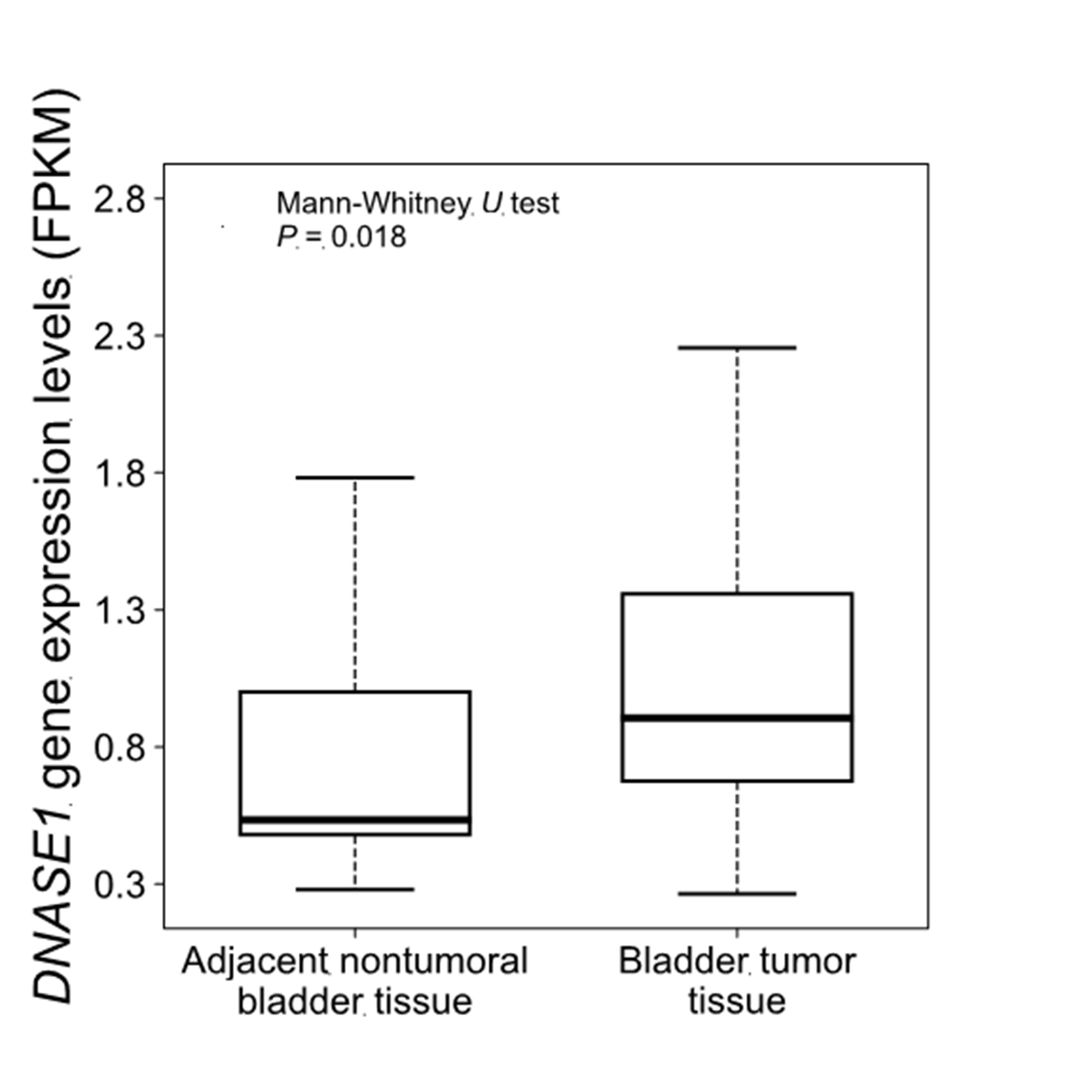

Supplement: S4 Fig — (TIF) [file pgen.1010262.s004.tif]

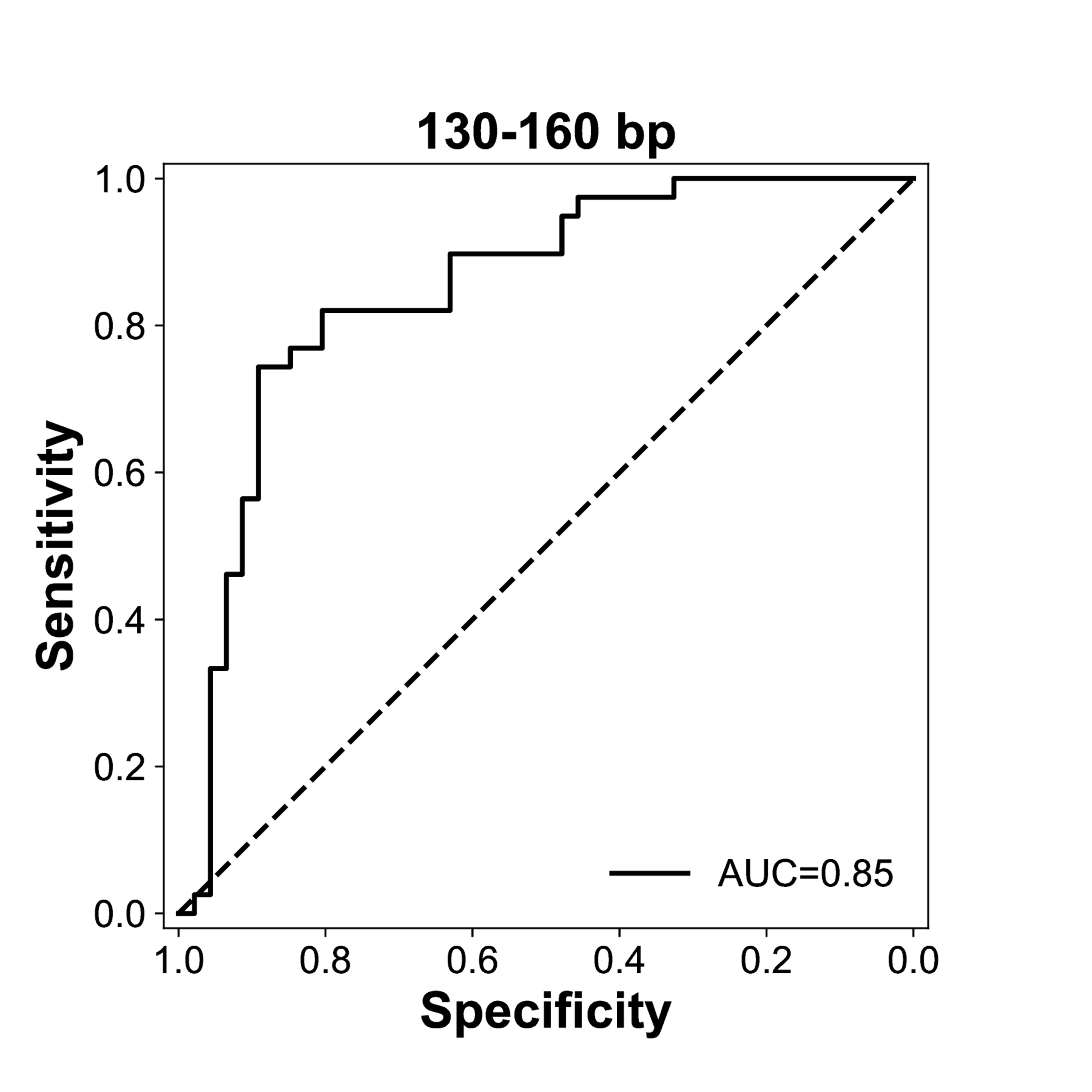

Supplement: S6 Fig — (TIF) [file pgen.1010262.s006.tif]

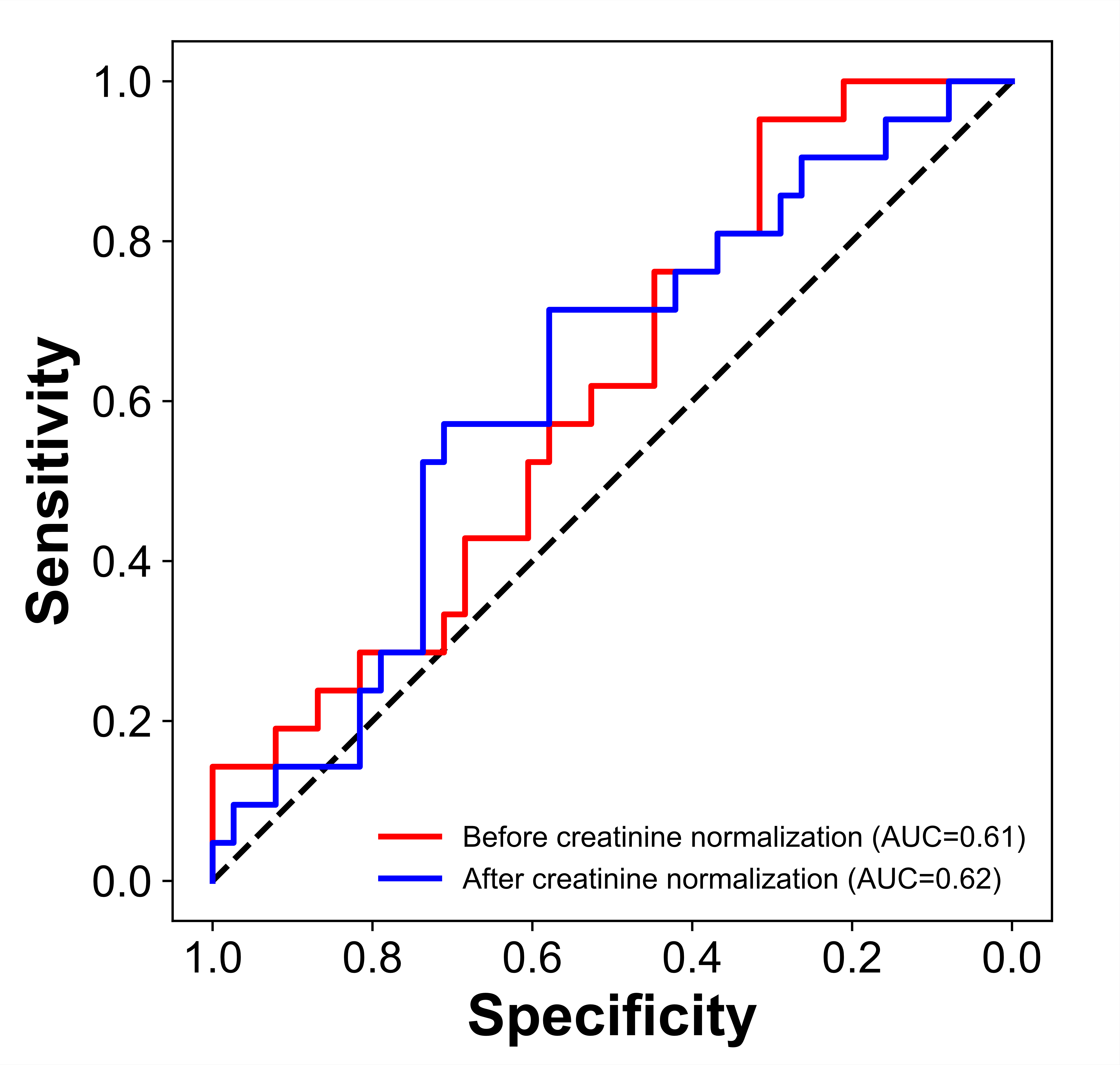

Supplement: S9 Fig — (TIFF) [file pgen.1010262.s009.tiff]
