# Supplementary material for: Fragmentomics of urinary cell-free DNA in nuclease knockout mouse models
Source: PLoS Genet. 2022 Jul 6;18(7):e1010262. doi: 10.1371/journal.pgen.1010262 (PMC9258866; doi:10.1371/journal.pgen.1010262)
Supplement: S1 Table — The metrics include the proportion of ucfDNA within DHSs, ucfDNA jaggedness, and ucfDNA end motifs. (DOCX) [file pgen.1010262.s010.docx]

S1_Table. The variation of the cutoff values for different metrics using leave-one-out cross-validation.

| **Metrics** | **Cutoff values for classification**  **between patients with and without bladder cancers** | | |
| --- | --- | --- | --- |
|  | **Median** | **Range** | **Coefficient of variation (CV)** |
| Proportion of ucfDNA within DHSs | 8.06 | 8.06 – 8.06 | < 0.01% |
| UcfDNA jaggedness | 31.64 | 31.01 – 32.02 | 0.33% |
| UcfDNA end motifs | 1.02 | 1.02 – 1.02 | 0.02% |
