# Supplementary material for: Fragmentomics of urinary cell-free DNA in nuclease knockout mouse models
Source: PLoS Genet. 2022 Jul 6;18(7):e1010262. doi: 10.1371/journal.pgen.1010262 (PMC9258866; doi:10.1371/journal.pgen.1010262)
Supplement: S2 Table — (DOCX) [file pgen.1010262.s011.docx]

S2_Table. Clinical information of the human subjects included in this study.

| Sample | Age | Gender | Group | Degree of invasiveness | ucfDNA concentration (GE/mL urine) | Creatinine concentration (mmol/L) |
| --- | --- | --- | --- | --- | --- | --- |
| T102 | 61 | M | CT | NA | 510 | 3.0 |
| T11 | 45 | M | CT | NA | 7038 | 3.0 |
| T16 | 46 | M | CT | NA | 390 | 10.6 |
| T29 | 54 | M | CT | NA | 1489 | 13.3 |
| T33 | 37 | F | CT | NA | 196 | 4.1 |
| T42 | 65 | M | CT | NA | 559 | 1.7 |
| T43 | 39 | M | CT | NA | 399 | 16.2 |
| T44 | 39 | M | CT | NA | 576 | 1.4 |
| T52 | 68 | M | CT | NA | 7506 | 11.2 |
| T63 | 25 | F | CT | NA | 20074 | 3.3 |
| T72 | 25 | F | CT | NA | 35671 | 5.8 |
| T85 | 61 | M | CT | NA | 225 | 4.3 |
| T86 | 51 | M | CT | NA | 559 | 8.1 |
| T94 | 30 | F | CT | NA | 2437 | 3.3 |
| T99 | 60 | M | CT | NA | 1249 | 10.3 |
| T10 | 50 | M | CT | NA | 974 | 18.2 |
| T18 | 30 | M | CT | NA | 2531 | 3.6 |
| T194 | 69 | M | CT | NA | 197 | 3.7 |
| T195 | 59 | F | CT | NA | 809 | 1.8 |
| T196 | 57 | M | CT | NA | 1315 | 2.2 |
| T19 | 53 | M | CT | NA | 2044 | 1.7 |
| T201 | 78 | F | CT | NA | 1154 | 0.7 |
| T20 | 53 | M | CT | NA | 25710 | 5.7 |
| T21 | 70 | F | CT | NA | 2830 | 3.0 |
| T34 | 60 | F | CT | NA | 1113 | 3.0 |
| T35 | 49 | F | CT | NA | 13088 | 8.3 |
| T40 | 50 | M | CT | NA | 1372 | 6.0 |
| T45 | 51 | M | CT | NA | 376 | 6.4 |
| T53 | 35 | M | CT | NA | 1265 | 12.0 |
| T54 | 25 | F | CT | NA | 15033 | 4.4 |
| T56 | 25 | F | CT | NA | 18211 | 4.0 |
| T57 | 35 | M | CT | NA | 1342 | 8.2 |
| T58 | 68 | M | CT | NA | 39415 | 10.5 |
| T59 | 68 | M | CT | NA | 52933 | 7.3 |
| T74 | 30 | M | CT | NA | 2406 | 6.5 |
| T75 | 50 | M | CT | NA | 1401 | 10.8 |
| T80 | 50 | M | CT | NA | 2919 | 13.5 |
| T159 | 68 | F | CT | NA | 1483 | 6.5 |
| TBR532 | 76 | F | CT | NA | 3482 | NA |
| TBR579 | 63 | M | Case | NMIBC LG | 380 | NA |
| TBR425 | 96 | F | Case | NMIBC LG | 731 | NA |
| T132 | 84 | F | Case | NMIBC LG | 855 | 2.2 |
| TBR419 | 73 | M | Case | NMIBC LG | 2349 | NA |
| TBR644 | 57 | M | Case | NMIBC LG | 773 | NA |
| TBR415 | 75 | F | Case | NMIBC LG | 275 | NA |
| T190 | 72 | F | Case | NMIBC LG | 353 | 4.4 |
| T139 | 78 | M | Case | NMIBC LG | 2881 | 18.2 |
| TBR445 | 66 | F | Case | NMIBC LG | 736 | NA |
| TBR679 | 76 | M | Case | NMIBC LG | 1568 | NA |
| TBR542 | 81 | M | Case | NMIBC LG | 151260 | NA |
| T179 | 77 | M | Case | NMIBC LG | 1491 | 1.6 |
| TBR494 | 69 | M | Case | NMIBC LG | 958 | NA |
| T145 | 62 | F | Case | NMIBC LG | 6790 | 21.2 |
| TBR633 | 57 | M | Case | NMIBC LG | 1034 | NA |
| TBR540 | 77 | F | Case | NMIBC LG | 4143 | NA |
| T146 | 81 | F | Case | NMIBC LG | 1636 | 12.5 |
| TBR728 | 84 | M | Case | NMIBC HG | 1837 | NA |
| T120 | 77 | F | Case | NMIBC HG | 406 | 3.0 |
| TBR538 | 80 | M | Case | NMIBC HG | 2870 | NA |
| TBR685 | 75 | M | Case | NMIBC HG | 35057 | NA |
| TBR441 | 91 | F | Case | NMIBC LG | 10313 | NA |
| TBR732 | 59 | M | Case | NMIBC LG | 4817 | NA |
| TBR1875 | 58 | M | Case | NMIBC HG | 2032 | 7.5 |
| TBR416 | 61 | M | Case | NMIBC HG | 978 | NA |
| TBR413 | 65 | M | Case | NMIBC HG | 275 | NA |
| TBR643 | 80 | F | Case | NMIBC HG | 4749 | NA |
| T134 | 58 | F | Case | NMIBC HG | 2983 | 5.1 |
| T158 | 71 | M | Case | NMIBC HG | 4096 | 4.4 |
| T175 | 59 | M | Case | NMIBC HG | 8170 | 8.5 |
| TBR406 | 85 | M | Case | NMIBC HG | 5024 | NA |
| TBR702 | 66 | M | Case | NMIBC HG | 7638 | NA |
| T138 | 60 | F | Case | NMIBC HG | 1279 | 2.0 |
| T154 | 69 | F | Case | NMIBC HG | 4589 | 1.4 |
| T171 | 76 | M | Case | NMIBC HG | 3917 | 12.5 |
| T188 | 91 | M | Case | NMIBC HG | 13278 | 1.8 |
| TBR561 | 64 | M | Case | NMIBC HG | 10765 | NA |
| TBR1848 | 54 | M | Case | MIBC HG | 13432 | 3.5 |
| TBR701 | 62 | M | Case | MIBC HG | 1883 | NA |
| T150 | 88 | M | Case | MIBC HG | 77422 | 6.0 |
| TBR1824 | 77 | M | Case | MIBC HG | 33586 | 5.8 |
| TBR687 | 73 | M | Case | MIBC HG | 19518 | NA |
| T22 | 73 | M | Case | MIBC HG | 680 | 1.1 |
| T23 | 57 | M | Case | MIBC HG | 1166 | 5.6 |
| T170 | 59 | M | Case | MIBC HG | 50808 | 6.3 |
| TBR447 | 70 | M | Case | MIBC HG | 2793 | NA |

CT: control. NMIBC LG: low-grade non–muscle invasive bladder cancer. NMIBC HG: high-grade non–muscle invasive bladder cancer. MIBC: muscle-invasive bladder cancer.
