# Supplementary material for: Fragmentomics of urinary cell-free DNA in nuclease knockout mouse models
Source: PLoS Genet. 2022 Jul 6;18(7):e1010262. doi: 10.1371/journal.pgen.1010262 (PMC9258866; doi:10.1371/journal.pgen.1010262)
Supplement: S3 Table — (DOCX) [file pgen.1010262.s012.docx]

| **Group** | **Sample ID** | **Mapped reads** |
| --- | --- | --- |
| **WT** | U4 | 49,558,626 |
| **WT** | U18 | 9,431,728 |
| **WT** | U19 | 50,235,533 |
| **WT** | U21 | 32,894,729 |
| **WT** | U30 | 44,528,346 |
| **WT** | U40 | 21,570,674 |
| **WT** | U41 | 51,189,633 |
| **WT** | U44 | 27,954,802 |
| **WT** | U51 | 10,835,562 |
| ***Dnase1l3*^-/-^** | U36 | 18,133,815 |
| ***Dnase1l3*^-/-^** | U65 | 31,158,557 |
| ***Dnase1l3*^-/-^** | U66 | 45,134,718 |
| ***Dnase1*^-/-^** | U2 | 41,183,976 |
| ***Dnase1*^-/-^** | U8 | 48,992,098 |
| ***Dnase1*^-/-^** | U24 | 34,950,786 |
| ***Dnase1*^-/-^** | U26 | 68,268,832 |
