# Supplementary material for: Fragmentomics of urinary cell-free DNA in nuclease knockout mouse models
Source: PLoS Genet. 2022 Jul 6;18(7):e1010262. doi: 10.1371/journal.pgen.1010262 (PMC9258866; doi:10.1371/journal.pgen.1010262)
Supplement: S4 Table — (DOCX) [file pgen.1010262.s013.docx]

| **Group** | **Sample ID** | **Mapped reads** |
| --- | --- | --- |
| **WT** | U20 | 19,379,473 |
| **WT** | U39 | 7,412,071 |
| **WT** | U47 | 18,185,871 |
| **WT** | U56 | 16,543,584 |
| ***Dnase1l3*^-/-^** | U36 | 10,260,732 |
| ***Dnase1l3*^-/-^** | U37 | 9,715,586 |
| ***Dnase1l3*^-/-^** | U46 | 7,589,355 |
| ***Dnase1*^-/-^** | U1 | 22,758,610 |
| ***Dnase1*^-/-^** | U3 | 19,673,241 |
| ***Dnase1*^-/-^** | U9 | 21,922,735 |
| ***Dnase1*^-/-^** | U15 | 28,919,775 |
| ***Dnase1*^-/-^** | U25 | 22,472,540 |
| ***Dnase1*^-/-^** | U33 | 19,421,500 |
